# The impact of masculinity beliefs and negative perceptions of aging on older adult men’s depression

**DOI:** 10.1093/geroni/igaf122.4214

**Published:** 2025-12-31

**Authors:** Darby Mackenstadt, David Buys, Mary Dozier, Carolyn Adams-Price

**Affiliations:** Mississippi State University, Durham, North Carolina, United States; Mississippi State University, Starkville, Mississippi, United States; Mississippi State University, Starkville, Mississippi, United States; Mississippi State University, Starkville, Mississippi, United States

## Abstract

Stereotype embodiment theory suggests how older adults that hold negative aging stereotypes may come to internalize them and begin to think negatively about their aging. Those who hold negative aging stereotypes also have more negative outcomes in physical and mental health. This project aimed to evaluate the relationship between masculinity standards, negative perceptions of aging, and depression in men aged 55 and older (N = 438) living in the southern United States. Results indicated that viewing aging as associated with psychosocial loss was associated with increased depression symptoms (β = .28, p < .001). Stereotypical masculine behaviors were not significantly associated with depression symptoms (β = -.08, p =.59); however, engaging in stereotypical masculine behaviors significantly moderated the effect of viewing aging as psychosocial loss on depression symptoms (β = .31, p = .04). Results suggest that participants who reported a high adherence to masculinity beliefs in combination with a negative perception of aging had higher symptoms of depression. Older adult men who have more negative perspectives of aging as well as participate more in traditional masculine behaviors may be at a higher risk for depression. Mental healthcare providers may benefit from recognizing the relationship between masculinity and negative perceptions of aging, and discussing thoughts or experiences related to aging with their clients who identify as men. Individuals who have a rigid adherence to hegemonic masculinity standards and who perceive aging negatively may benefit from discussing these views, and how to acknowledge the positive aspects of growing older.

